# CircRNA circ_0124554 blocked the ubiquitination of AKT promoting the skip lymphovascular invasion on hepatic metastasis in colorectal cancer

**DOI:** 10.1038/s41419-021-03565-3

**Published:** 2021-03-15

**Authors:** Junwei Tang, Chuan Zhang, Yuanjian Huang, Lu Wang, Ziwei Xu, Dongsheng Zhang, Yue Zhang, Wen Peng, Yifei Feng, Yueming Sun

**Affiliations:** Colorectal Surgery Division, Department of General Surgery, The First Affiliated Hospital of Nanjing Medical University, Nanjing, Jiangsu People’s Republic of China

**Keywords:** Colon cancer, Oncogenes

## Abstract

Colorectal cancer (CRC) is the fourth most common cancer in men and the third most common cancer in women worldwide. The incidence and mortality of CRC was increasing rapidly in China. Lymph node-negative colorectal cancer patients with synchronous liver metastasis (LNLM1) was defined as “skip” lymph vascular invasion on hepatic metastasis, who presenting poor prognosis. We aiming to investigate the potential mechanism for the “skip” lymph vascular invasion on hepatic metastasis in colorectal cancer. The microarray was applied for screening the transcription landscape of circRNA in lymph node negative CRC patients with synchronous liver metastasis (LNLM1) or without liver metastasis (LNLM0). We identified the aberrant increased circRNA circ_0124554 (also entitled as circ-LNLM) in tumor tissues of LNLM1 patients comparing with either the tumor tissues of LNLM0 or adjacent tissues of LNLM1. Circ-LNLM1 expression was highly correlated with liver metastasis and vascular invasion. Ectopic expression of cytoplasmic located circ-LNLM could promote invasion of CRC cells and induced the liver metastasis in animal models through the direct binding with AKT. The phosphorylation of AKT (T308/S473) was activated due to the blocked ubiquitination site of Lys in 0-52aa peptide of circ-LNLM. Endogenous plasma expression of circ-LNLM induced poor prognosis of LNLM1 and could distinguish LNLM1 patients from LNLM0. In conclusion, the circ-LNLM blocked the ubiquitination of AKT could promote the early metastasis especially for the lymph node-negative colorectal cancer patients with synchronous liver metastasis. The circ-LNLM might be prognosis and diagnosis biomarker for LNLM1 patients.

## Introduction

According to the Cancer Statistics in 2020, colorectal cancer (CRC) is one of the most common gastrointestinal malignancies, ranking the third in males and the second in females, with high incidence, difficult treatment and poor prognosis^1,2^. Worldwide, about 1.8 million new cases of colorectal cancer occur each year, and about 880,000 colorectal cancer deaths occurred^3^. In developed countries such as Europe and America, the incidence and mortality of colorectal cancer have reached the third and second place among all cancer types^4^. In China, with the improvement of people’s living standards and the change of diet structure, the incidence and mortality of colorectal cancer are increasing year by year. In combination with China’s specific national conditions, the rate of early diagnosis and treatment of colorectal cancer is much lower than that in developed countries such as Europe, America and Japan^5^. Most of the patients are diagnosed with local advanced stage or distant metastasis, especially liver metastasis, with poor treatment effect and poor prognosis.

Previous studies have shown that about 15–25% of patients with colorectal cancer have synchronous liver metastasis, the most common organ with distant colorectal cancer metastasis, while as many as 20–25% of patients will have synchronous liver metastasis after primary resection of colorectal cancer^6,7^. The prognosis of patients with liver metastasis is very poor. Without any treatment, the natural survival is about 6.6 months, the 3-year survival rate is 5%, and the 5-year survival rate is less than 1%^8^. The main reason is that when liver metastasis is found, the resection rate of liver metastasis foci is not high, the patients have a late stage of disease, and the disease progresses rapidly. Therefore, in order to improve the treatment effect and prognosis of patients with colorectal cancer survival, explore early metastasis of colorectal cancer development of specific molecular mechanism, and, in turn, screening effective drug targets, reducing colorectal cancer metastasis and improving the effect of the therapy of colorectal cancer, it is of great clinical significance and research value.

Although lymph node metastasis is the most common mode of colorectal cancer metastasis, lymph node metastasis also plays an important role in predicting the prognosis of colorectal cancer patients. In addition, colorectal cancer patients with synchronous liver metastasis also have positive postoperative lymph node metastasis, but in clinical work, we gradually found that lymph node negative colorectal cancer patients at the initial diagnosis of synchronous liver metastasis. Combined with the colorectal cancer metastasis pathway, this type of lymph node negative colorectal cancer with simultaneous liver metastasis, the cancer cells may have been transferred to the liver by blood route. This is mainly decided by the anatomical features: portal vein system exists between intestinal blood and liver vascular network, transferred to the liver cancer cells through the portal system, and early in the lymphatic system transfer, prompt such there may be a more aggressive tumor biology function, and there is evidence that such relative to lymph node negative patients without hepatic metastasis in patients with worse prognosis^9,10^. Therefore, the in-depth exploration of the tumor biological function of these patients, the analysis of the specific mechanism of simultaneous liver metastasis in lymph node negative colorectal cancer patients, and the search for key targets to promote the progression of tumor invasion can provide an important theoretical basis for the subsequent development of liver metastasis targets for colorectal cancer.

Circular RNA, also known as circRNA, was first discovered in RNA viruses in 1976. In recent years, with the rapid development of high-throughput sequencing and other technologies, a large number of circRNAs have been discovered and their biological roles have been gradually investigated^11^. CircRNAs can be classified into three categories according to their source: intron circRNAs, exon circRNAs, and circRNAs formed by a mixture of introns and exons^12^. CircRNA is widely distributed in cells with time-specific, tissue-specific, and disease-specific. CircRNA is widely distributed in tissues and highly conserved in structure^13,14^. Due to the absence of poly(A) tail structure and cap structure, circRNA is highly stable to nucleic acid exonuclease and extremely stable to cells in tissues and exosomes^15^. Therefore, combined with clinical practice.

In order to deeply analyze the potential mechanism of liver metastasis of colorectal cancer, this study took lymph node negative with liver metastasis (LNLM1) as the object of study: such patients had relatively poor prognosis and suggested that the tumor had strong invasion ability. We intended to screen for circRNAs that were highly associated with colorectal cancer metastasis through high-throughput techniques, compared with lymph node negative without liver metastasis (LNLM0) in lymph node negative colorectal cancer patients. In order to avoid the error of metastasis event caused by tumor invasion of other adjacent organs, according to TNM stage, only patients with T stage T1-T3 were included in this study.

## Results

### Comparison of the circRNA transcriptomic profiles in LNLM0 and LNLM1

**Fig. 1:**
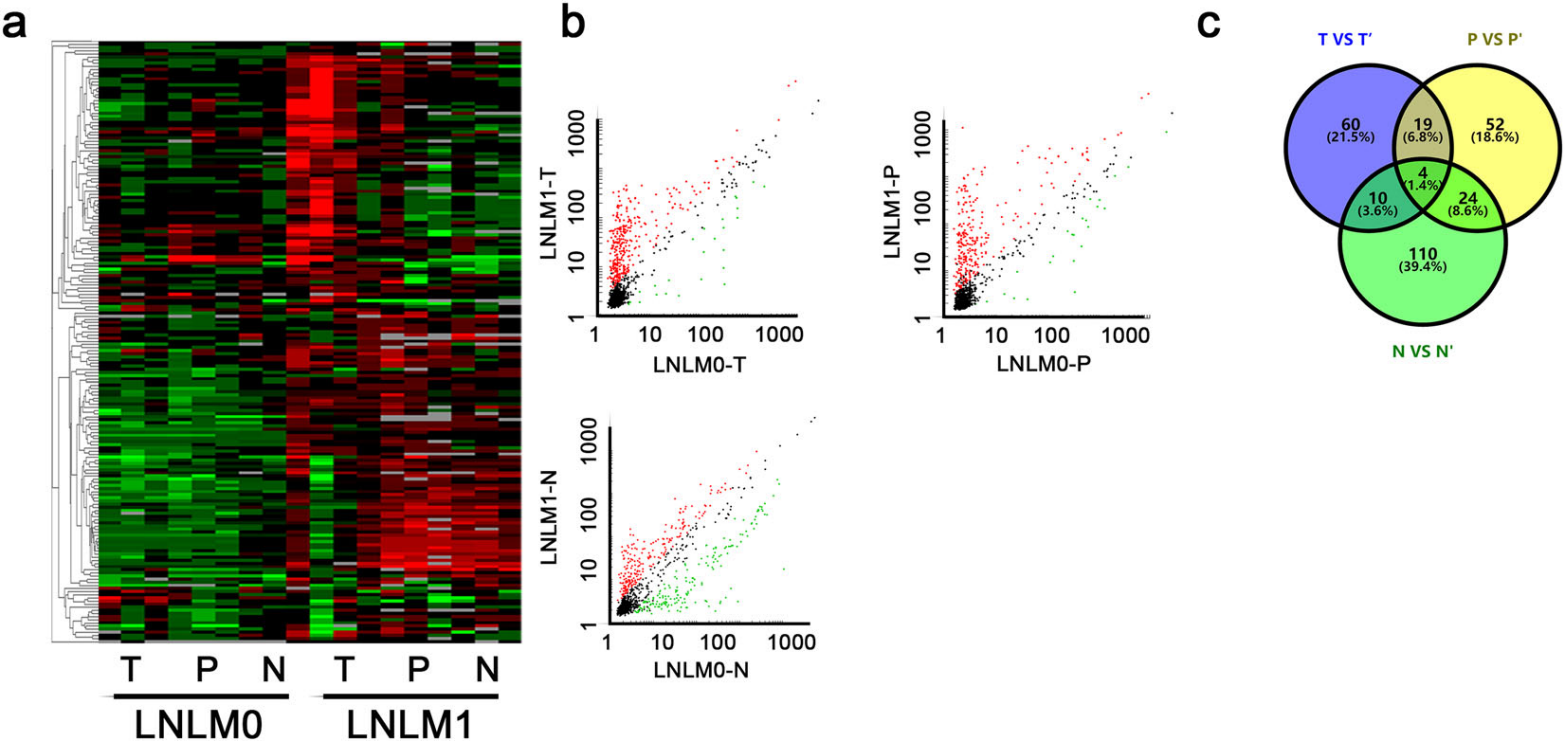
The abnormal expression landscape of circRNA in tissues of colorectal cancer. **a** Cluster analysis of different expressed cirRNAs. Red indicated upregulated while green indicated downregulated. **b** Scatter plot for circRNA in tumor tissues, adjacent tumor tissues and normal tissues of LNLM1 and LNLM0 patients. **c** Venny analysis for different expressed in three groups. LNLM1: lymph node negative CRC patients with synchronous liver metastasis; LNLM0: lymph node negative CRC patients without synchronous liver metastasis. T: tumor tissues of LNLM1, P: adjacent tumor tissues of LNLM1, N: normal tissues of LNLM1; T’: tumor tissues of LNLM0, P’: adjacent tumor tissues of LNLM0, N’: normal tissues of LNLM0.

In this study, Total RNA were extracted from the tumor tissues, corresponding adjacent tumor tissues and normal tissues from colorectal cancer patients with synchronous liver metastasis (LNLM1) or without liver metastasis (LNLM0). The total circRNA transcriptomic profiles was detected. A differential expression profile was obtained by comparing the microarray signals from the two groups, which showed that 289 circRNAs were differentially expressed in LNLM1 group with fold changes of 4/0.25. In an unsupervised clustering analysis of all the transcripts, we detected significant differences in the expression signatures of the three sets of samples (Fig. 1a). The separated analysis was further used in the three sets, in brief, circRNAs with significant difference expression was compared in tumor tissues, adjacent tumor tissues and normal tissues, respectively. As presented in Fig. 1b, the scatter plots showed total 93 circRNAs were increased in tumor tissues of LNLM1 patients, 99 circRNAs in adjacent tumor tissues of LNLM1 patients were elevated while 148 circRNAs were selected in normal tissues. To identify the candidate circRNAs, we draw the venny analysis. Total four circRANs including hsa_circ_0009582, hsa_circ_0079875, hsa_circ_0124554 and hsa_circ_0097743 showed consistent higher expression in all tissues of LNLM1 patient (Fig. 1c). We intended to use the four circRNAs as candidate targets for further validation.

### Expression of hsa_circ_0124554 was significantly upregulated in LNLM1 tissues

**Fig. 2:**
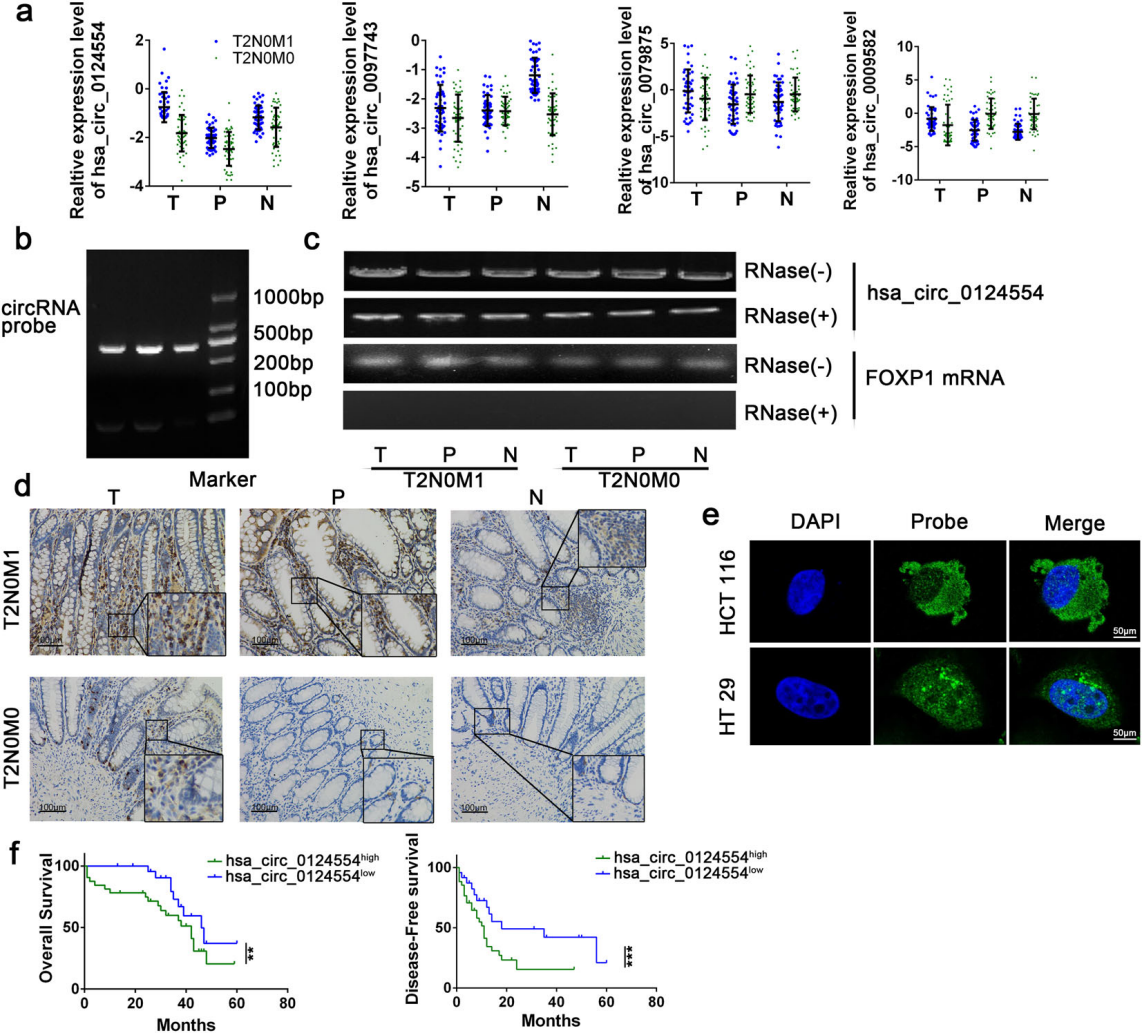
Upregulated hsa_circ_0124554 predicted worse prognosis of CRC. **a** Relatively increased levels hsa_circ_0124554 and other three candidate circRNAs. **b** RNAs were isolated from tissues of CRC patients and subjected to a northern blot analysis using a probe specific for hsa_circ_0124554. **c** Standard PCR was performed to detect the hsa_circ_0124554 from control RNA or digested RNAs using RNase R exonuclease LNLM1 patient and LNLM0 patient. **d** The subcellular location and intensity of hsa_circ_0124554 were examined with in situ hybridization in sections from LNLM1 patients and LNLM0 patients. **e** The subcellular location of hsa_circ_0124554 were detected by IF with 400X magnification. **f** Kaplan–Meier survival curves of overall survival and disease-free survival based on hsa_circ_0124554 expression in CRC patients. All patients were divided into two groups based on the median level of hsa_circ_0124554. The log-rank test was used to calculate the significant level. Data are presented as means ± SD. LNLM1: lymph node negative CRC patients with synchronous liver metastasis; LNLM0: lymph node negative CRC patients without synchronous liver metastasis. T: tumor tissues of LNLM1, P: adjacent tumor tissues of LNLM1, N: normal tissues of LNLM1.

We examined these investigated circRNAs candidates in tumor tissues, adjacent tumor tissues and paired normal tissue samples from 40 LNLM1 patients and 40 LNLM0 patients. Results confirmed that hsa_circ_0124554 was consistent with the chip expression in all three groups, hsa_circ_0097743 was not statistically significant in the paracancer tissues, and hsa_circ_000958 and hsa_circ_0079875 were contrary to the chip expression in the paracancer group and normal tissues (Fig. 2a). Next, the totaled RNA extracted from three patients with LNLM1 and three patients with LNLM0 was subjected to northern blot using a probe specific for hsa_circ_0124554, we found that hsa_circ_0124554 was existed in human colorectal tissues (Fig. 2b). Has_circ_0124554 was located at chr3:71090478-71096246 and range from exon 9,10 of the FOXP1 genome (UCSC data in NCBI). Meanwhile, the RNase exonuclease was applied to test the stable expression of circRNA. We found that hsa_circ_0124554 was resistant to RNase after digestion of RNA. The FOXP1 (linear form) was used as negative control (Fig. 2c). In situ hybridization (ISH) by using a biotin-labeled specific probe were further carried out to measure hsa_circ_0124554 expression. Results indicated that more hsa_circ_0124554 was in the tissues of LNLM1 patients while the tumor tissues presented the highest stain compared with the adjacent tumor tissues and normal tissues (Fig. 2d). By using fluorescence in situ hybridization (FISH) assay in cell lines of CRC including HCT 116 and HT 29, we noted that hsa_circ_0124554 was mainly localized in cytoplasm and nucleus but was predominately enriched in the cytoplasm in cells (Fig. 2e).

To further investigate the relationship between hsa_circ_0124554 level and clinical characteristics, we analyzed the expression hsa_circ_0124554 in different group according to the clinical information. We found that in the two groups, no difference in age, gender, T stage, tumor differentiation and pathological type was found, but the LNLM1 patients presented probability higher vascular invasion (Supplementary Table 1). Next, by using the medium expression of hsa_circ_0124554 in tumor tissues as cutoff, we divided the patients into hsa_circ_0124554^high^ and hsa_circ_0124554^low^ group. We found higher expression of hsa_circ_0124554 was correlated with vascular invasion and liver metastasis, no correction was found in T stage, tumor differentiation or pathological types indicating that its transfer may be associated with tumor cells into the bloodstream (Supplementary Table 2). We also investigated the correlation of hsa_circ_0124554 expression with the prognosis of all CRC patients including LNLM1 and LNLM0 groups. We found that CRC patients with higher hsa_circ_0124554 expression showed a poorer prognosis by Kaplan–Meier survival analysis (Fig. 2f). Taken together, our data suggest that hsa_circ_0124554 is highly expressed in LNLM1 tissues and correlated with a poor prognosis.

### Increased hsa_circ_0124554 promoted metastasis of CRC

**Fig. 3:**
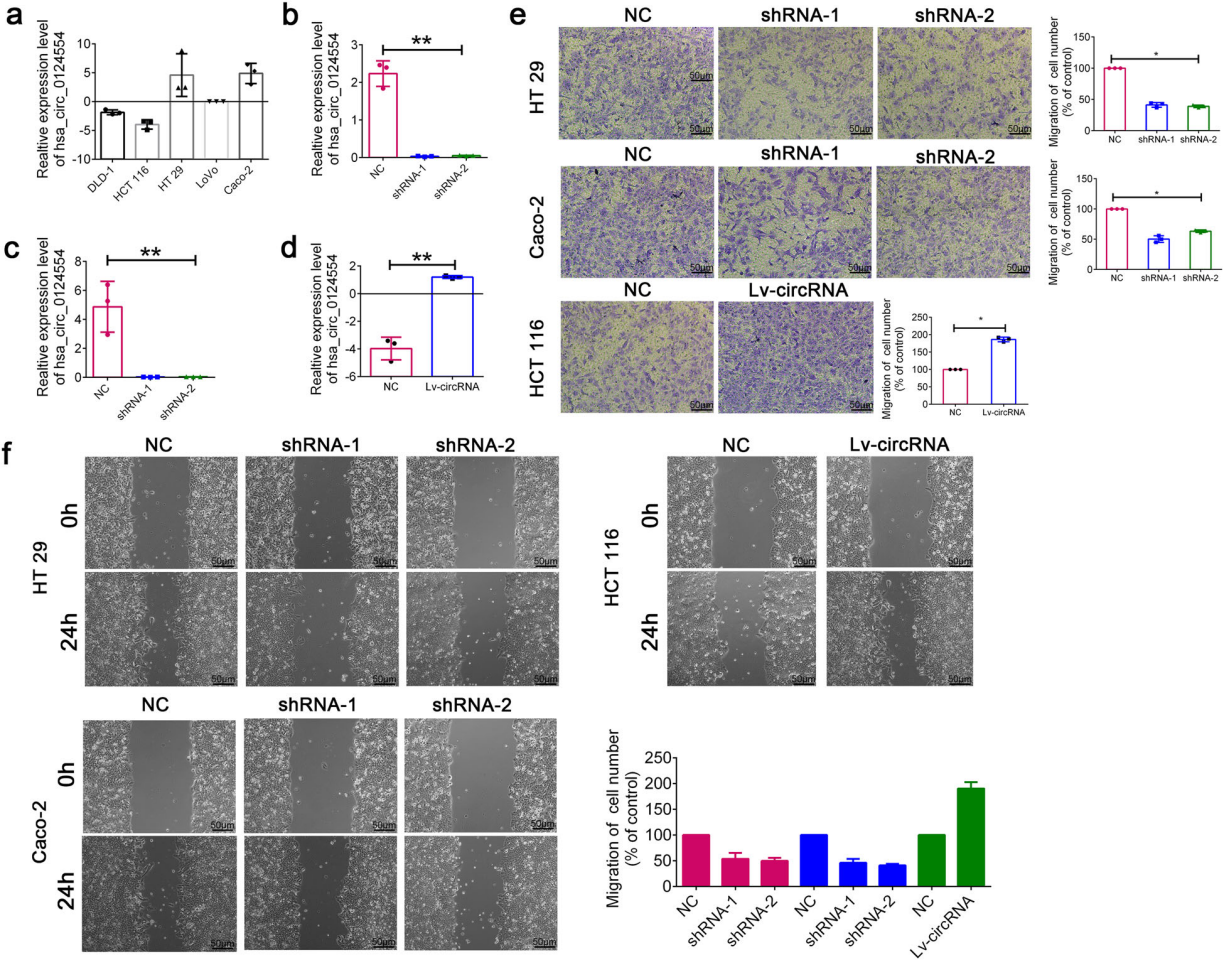
Hsa_circ_0124554 promoted invasion of CRC cells. **a** Relative expression of hsa_circ_0124554 in CRC cell lines. **b** Relative expression of hsa_circ_0124554 in HT 29 cell lines treated with shRNA. **c** Relative expression of hsa_circ_0124554 in Caco-2 cell lines treated with shRNA. **d** Relative expression of hsa_circ_0124554 in HCT 116 cell lines treated with overexpression lentivirus. **e** Transwell assay in cells treated with shRNAs and overexpression lentivirus. **f** Would healing assay in cells treated with shRNAs and overexpression lentivirus. Data are presented as means ± SD (*P < 0.05, **P < 0.01).

We next investigated the function role of hsa_circ_0124554 in CRC cell lines. Firstly, the endogenous expression of hsa_circ_0124554 was detected in CRC cell lines. The results demonstrated that hsa_circ_0124554 was overexpressed in CRC cell lines. Decreased level of hsa_circ_0124554 was obtained in HCT 116 cells while increased level was found in HT 29 and Caco-2 (Fig. 3a). To further confirm the circular characteristics of hsa_circ_0124554, Actinomycin D, an inhibitor of transcription that degraded FOXP1 mRNA was added in HT29, Cacao2 and HCT116 cells. Evidently, hsa_circ_0124554 was more stable and resistant to Actinomycin D treatment (Supplementary Fig. 1a). Subsequently, the head-to-tail back splicing was validated in the RT–PCR product of hsa_circ_0124554 with Sanger sequencing (Supplementary Fig. 1b). We next designed the knock-down lentivirus in HT 29 and Caco-2. The suppression level of hsa_circ_0124554 was confirmed (Fig. 3b, c). The overexpression lentivirus was applied and validated in HCT 116 (Fig. 3d). The knock-down lentivirus and the overexpression lentivirus were all specifically targeted at hsa_circ_0124554, but not its linear form, FOXP1 mRNA (Supplementary Fig. 1,c, d). The transwell assay was employed to examine the loss-and gain-of-function of hsa_circ_0124554. As presented in Fig. 3e, hsa_circ_0124554 knockout significantly suppressed the invasion of CRC cells while ectopic expression promoted the invasion. Meanwhile, would healing assay also indicated that when the expression of hsa_circ_0124554 was inhibited, the migration ability of cells was weakened, while when the expression of hsa_circ_0124554 was enhanced, the migration ability was significantly increased compared with the control group (Fig. 3f). We also detected whether the loss or gain of hsa_circ_0124554 could affect cell proliferation, apoptosis, or cell cycle. We also employed another group by knocking down or overexpressing the linear form of circ_0124554. As presented in Supplementary Fig. S2, we found that the invasion of tumor cells was promoted by the circular form instead of the linear form. Functional experiments from CCK8 and FACS results demonstrated that no difference was obtained in cell proliferation, cell cycle or apoptosis with hsa_circ_0124554 in CRC cells (Supplementary Fig. S3).

### Hsa_circ_0124554 could direct bind with AKT protein

**Fig. 4:**
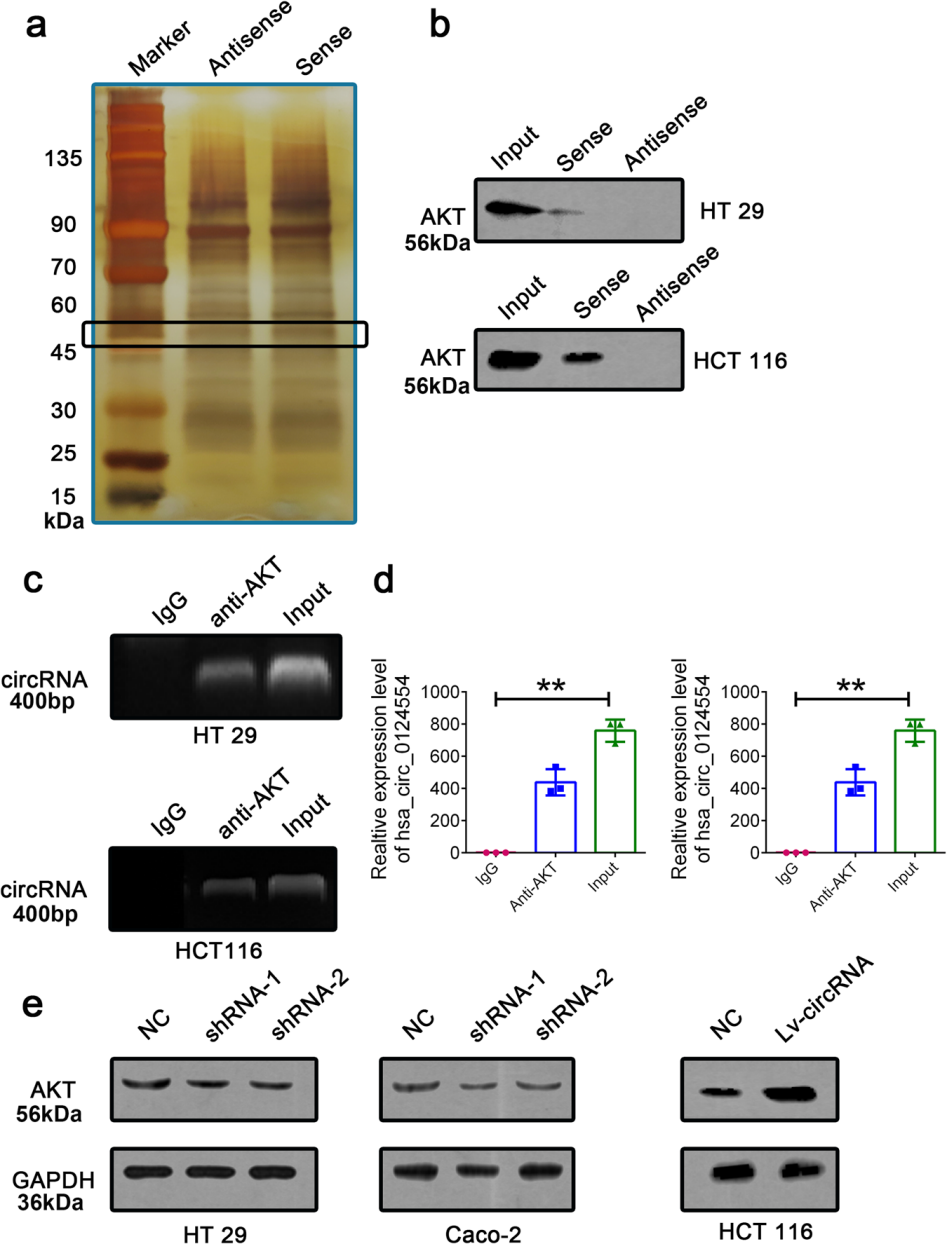
Hsa_circ_0124554 active AKT through directly binding. **a** RNA pull-down assay was conducted in cells with hsa_circ_0124554 sense and antisense. **b** Western blot analysis with anti-AKT antibody indicated the existence of AKT within the hsa_circ_0124554 sense RNA probe pull-down samples in HT 29 and HCT 116 cells. **c** RIP assay was performed using an anti-AKT antibody and was confirmed with agarose gel electrophoresis using a different probe. **d** Fold increases of RIP were calculated by comparison with the input. **e** Protein expression of AKT in cells with hsa_circ_0124554 overexpression or suppression. Data are presented as means ± SD (**P < 0.01).

We further investigated the mechanisms by which hsa_circ_0124554 promoted the invasion in CRC cells. Recent evidence indicated that circRNAs could involve in molecular regulation through interacting with certain proteins. Based on this hypothesis, we identified potential target which may interact with hsa_circ_0124554 by performing the RNA pull-down assay in HCT 116 cells by using the biotin labeled probe of hsa_circ_0124554. The hsa_circ_0124554-related proteins were determined using SDS/PAGE and silver staining (Fig. 4a). By using mass spectrometry, potential proteins were identified between 45 kDa and 60 kDa compared with the antisense group. Among the annotated protein, we found the AKT protein with the highest score. Western blot analysis with anti-AKT antibody indicated the existence of AKT within the hsa_circ_0124554 sense RNA probe pull-down samples in HT 29 and HCT 116 cells (Fig. 4b). We further performed the RIP assay to check whether AKT could recruit hsa_circ_0124554 by using AKT specific antibody in HT 29 and HCT 116 cells. By detecting the enriched RNA, the amplification band of hsa_circ_0124554 was obtained by agarose electrophoresis after PCR (Fig. 4c, d and Supplementary Fig. 4). Moreover, we observed that knockdown of endogenous hsa_circ_0124554 decreased the protein expression of AKT in HT 29 and HCT 116 cells, while increased hsa_circ_0124554 induced the expression of AKT in Caco-2 cells (Fig. 4e).

### Cytoplasmic hsa_circ_0124554 enhances the phosphorylation of AKT by preventing its ubiquitination

**Fig. 5:**
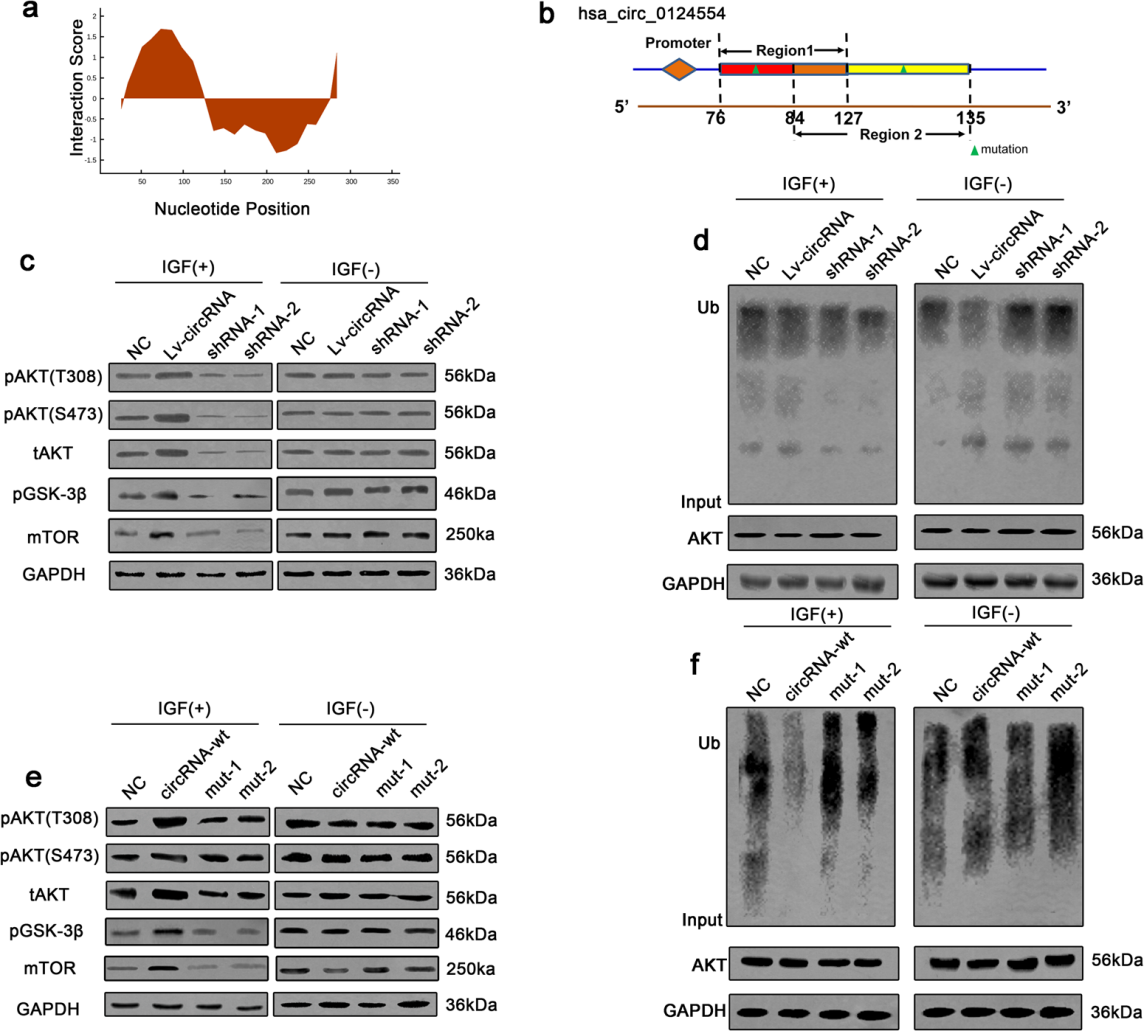
Hsa_circ_0124554 enhances the phosphorylation and prevents the ubiquitination of AKT. **a** The predicted binding region of hsa_circ_0124554 with AKT. **b** The schematic map of mutant sites. **c** The phosphorylation of AKT and downstream signaling were detected with IGF1 stimulation. **d** Ubiquitination of AKT in CRC cells treated with different groups in the presence of IGF1. **e** The phosphorylation of AKT and downstream signaling were detected with IGF1 stimulation. mut1 indicated mutation in region1, mut2 indicated mutation in region 2. **f** Ubiquitination of AKT in CRC cells treated with different groups in the presence of IGF1. mut1 indicated mutation in region1, mut2 indicated mutation in region 2.

Based on these data, the bioinformatic software catRAPID (http://service.tartaglialab.com) was used to predict the binding region in detail. As shown in Fig. 5a, the main regions for hsa_circ_0124554 interacted with AKT was predicted. The detailed two region located in hsa_circ_0124554 were 76-127nt and 84-135nt (Supplementary Fig. 5a) and the interaction matrix between protein and nucleotide indicated that the 01-55aa peptide of AKT was the target region (Supplementary Fig. 5b). The matched mutation in the two regions in hsa_circ_0124554 was designed and cloned into target cells (Fig. 5b).

The AKT protein size is about 60kD, with the N-terminal to C-terminal domains being the PH domain, the kinase/catalytic domain, and the regulatory domain. In this region, there are six AKT ubiquitination sites: K6, K11, K27, K29, K33, K48 and the adjacent ubiquitination site K63^16^. Ubiquitination of K48 is mediated by the 26 S proteasome^17^, which leads directly to the degradation of AKT protein, while ubiquitination of K63 plays a key role in AKT signaling and protein transport^18^. Although the role of other types of ubiquitination is unknown, recent studies suggest that they may also be involved in triggering protein degradation. In combination with the above predicted results, the AKT Rate limiting component (ubiquitination site) was found in the 0-52aa amino acid site of AKT, so the team speculated that the interaction between hsa_circ_0124554 and AKT might involve the regulation of the ubiquitination level of AKT. By adding IGF1 as stimulator, we found the phosphorylation of AKT, downstream of AKT including GSK-3β and mTOR (both T308 and S473) and total expression of AKT was enhanced with hsa_circ_0124554 overexpression while could be attenuated by silencing of hsa_circ_0124554. No difference was identified without treating with IGF (Fig. 5c, Supplementary Fig. 6). As the ubiquitination site was predicted in AKT, we further investigated the ubiquitination level of AKT in different condition descripted above. Interestingly, the ubiquitination of AKT was suppressed in hsa_circ_0124554 overexpressed cells while could be upregulated with hsa_circ_0124554 knocking down (Fig. 5d, Supplementary Fig. 7). We next detected the phosphorylation and ubiquitination level of AKT in cells treated with mutation type of hsa_circ_0124554. As presented in Fig. 5e and Supplementary Fig. 6, hsa_circ_0124554 either region 1 or region 2 was mutant, was largely unable to increase or sustain the phosphorylation of AKT by IGF. Consistently, the ubiquitination level of AKT presented no alteration when overexpression of hsa_circ_0124554 with mutant in region 1 or 2 (Fig. 5f, Supplementary Fig. 7).

### Hsa_circ_0124554 enhances tumor metastasis in vivo

**Fig. 6:**
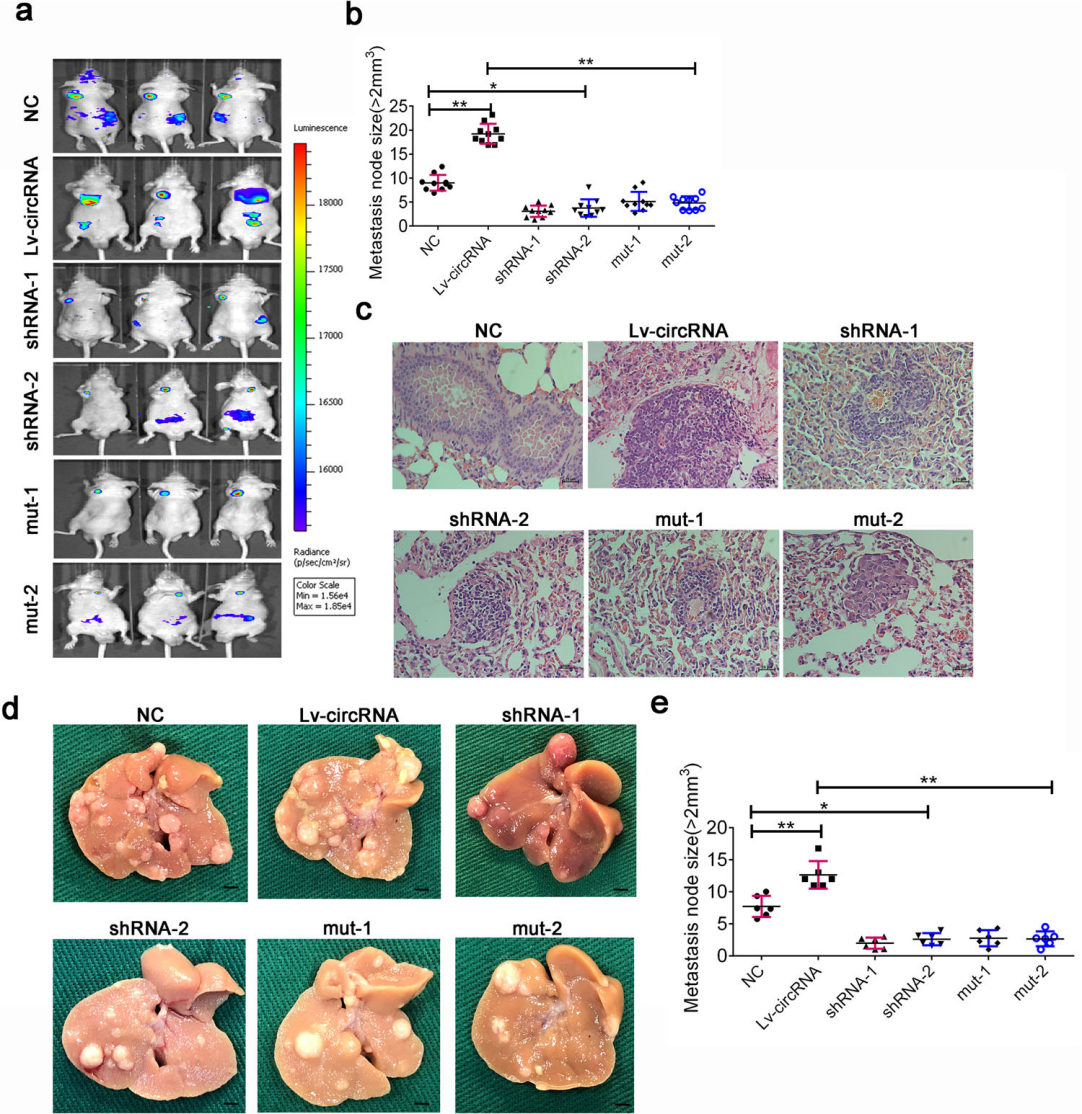
Hsa_circ_0124554 promoted the metastasis of CRC in vivo. **a** The lung metastasis node of mice was measure by IVIS Lumina II image system. **b** The lung metastasis node size in different groups. **c** H-E stain for the lung metastasis node. **d** Representative liver metastasis node in different groups. **e** The liver metastasis node size in different groups. Data are presented as means ± SD (*P < 0.05, **P < 0.01).

We next investigated the functional role of hsa_circ_0124554 and its integrated regulation with AKT in vivo with a mice tail vein xenograft model. Six groups each containing 10 mice were set up by injecting different treated Lovo cells from the aforementioned in vitro experiments, respectively. The metastasis was monitored by the IVIS Lumina II image system when the metastasis with volume >2 mm^3^ and was calculated and compared in each group. The results revealed that metastasis ability can be significantly enhanced in hsa_circ_0124554 overexpression group compared with the paired control group. Meanwhile, the suppression of hsa_circ_0124554 by shRNA technology could reduce the metastasis. Cells treated with the mutant type of hsa_circ_0124554 injected into mice presented a suppressed metastasis (Fig. 6a, b). The metastasis tumors in each group were obtained and analyzed by hematoxylin and eosin (H&E) staining, which presented similar results (Fig. 6c). We further investigated the function of Hsa_circ_0124554 activated the AKT associated signaling promoting the early metastasis through the liver metastasis model. As we could obtained from the model, with the increased of Hsa_circ_0124554 expression, the liver metastases were gradually aggravated, and the liver metastases were also decreased after knockdown of hsa_circ_0124554 or the specific mutant type of hsa_circ_0124554 (Fig. 6d, e).

## Discussion

The metastasis especially the liver metastasis is the main cause for poor prognosis of CRC^6,19^. Tumor metastasis is a complex cascade in which cancer cells acquire an aggressive phenotype in the primary site, migrate into the circulatory system, enter the metastatic palace, and begin secondary growth^20^.

Due to the conserved and stable characteristics, Numerous researchers focused on circRNA and its biological functions, which have become a hotspot in tumor research. Researchers have found that circular RNA can be a variety of mechanisms involved in gene expression and regulation: circRNA interacted with classic molecular miRNA, through the endogenous competitive RNA approach as the miRNA sponges, participating in the regulation of gene expression, also can be directly involved in the mRNA or protein and RNA transcription process, the formation of protein complexes, and affect gene expression^21,22^. In addition, some circRNA can serve as transcription template synthesis of protein. Currently, some circRNAs in colorectal cancer have been detected by high-throughput testing and confirmed to be involved in the progression of liver metastasis. Studies have shown that circPPP1R12A directly encoded PPP1R12A-C peptide participating in YAP signaling transduction in colorectal cancer^23^. Circ-NSD2 is involved in the occurrence of liver metastasis through the formation of circ-NSD2/miR-199b-5p/DDR1/JAG1 axis^24^. Liver metastasis of colorectal cancer has been regarded as a relatively simple biological event in current studies. The screening process in the study was based on comparisons with colorectal cancer patients without liver metastasis, resulting in differentially expressed circRNAs that lacked relative specificity and did not take lymph node metastasis into account.

In study, we mainly focused on the lymph node negative CRC patients with synchronous liver metastasis. The selected circRNA hsa_circ_0124554 presented a strong ability in promoting the invasion of CRC cells. Through the RNA pull-down screening, we anchored AKT as the target gene of hsa_circ_0124554, and combined with bioinformatics prediction and subsequent validation, finally found that hsa_circ_0124554 can directly bind to the degradation ubiquitination region of AKT, inhibit the ubiquitination degradation of AKT, and thus continue to activate the AKT signaling pathway. AKT is one of the important effector elements of the classical signaling pathway PI3K/AKT. The PI3K/AKT signaling pathway is considered to be a key pathway for tumor development and is ubiquitous in a variety of human tumors. AKT itself functions in an activated form of phosphorylation. When stimulated by specific ligands (e.g., IGF, insulin, etc.), AKT is phosphorylated at P13K, PDKl, and ILK at Thr308 in the catalytic domain and Ser473 in the regulatory domain. Only phosphorylation at these two sites can fully activate AKT. Activated AKT has been demonstrated to be highly expressed in many human cancers and to play a significant role in facilitating the migration, invasion, survival, and resistance of tumor cells. In this study, we found that hsa_circ_0124554 can specifically bind to AKT, reduce the degradation of AKTt by mediating the ubiquitination of AKT, and continue the continuous activation of downstream signaling pathways of Akt, including GSK-3β and mTOR, so as to generate events targeting the early metastasis of tumors.

Taken together, our study identifies a new circular RNA, termed hsa_circ_0124554 regulated in lymph node negative CRC patients with synchronous liver metastasis. Hsa_circ_0124554 directly binds with AKT, activates the AKT associated signaling, promotes the metastasis of tumor and indicates worse prognosis of CRC. Our data provide a platform for further elucidating mechanisms underlying the metastasis of CRC and identifying diagnostic or therapeutic targets.

## Materials and methods

### Patient samples

Eighty CRC patient samples were obtained from 2003–2017, including their tumor tissues, corresponding adjacent tumor tissues and normal tissues. The CRC patients was diagnosed by surgery and pathology in the First Affiliated Hospital of Nanjing Medical University. After sample collection, liquid nitrogen was frozen and transported, and stored in −70°C deep cryogenic refrigerator. Written consents were approved by the patients enrolled in this study. The clinical stage was based on the 8th edition of the International Union Against Cancer (UICC) on Tumor-Node-Metastasis (TNM) staging system. All experiments were performed in compliance with government policies and the Helsinki Declaration. The individuals were informed about the study and gave consent prior to the specimen collection. All experiments were approved by the ethics committee of the First Affiliated Hospital of Nanjing Medical University.

### High throughput detection

Total RNA from each sample was quantified using a NanoDrop ND-1000. The sample preparation and microarray hybridization were performed based on Arraystar’s standard protocols (KANGCHEN Biotech, Shanghai, China). In brief, total RNA was digested with RNase R (Epicentre, Inc.) to remove linear RNAs and enrich for circular RNAs. Then, the enriched circRNAs were amplified and transcribed into fluorescent cRNA using a random priming method (Arraystar Super RNA Labeling Kit; Arraystar). The labeled cRNAs were hybridized onto the Arraystar Human circRNA Array V2 (8 × 15 K, Arraystar). Next, the slides were washed and the arrays were scanned using an Agilent Scanner G2505C. Agilent Feature Extraction software (version 11.0.1.1) was applied to analyze the acquired array images. Quantile normalization and subsequent data processing were performed using the R software limma package. Differentially expressed circRNAs with statistical significance between two groups were identified through Volcano Plot filtering. Significantly differentially expressed circRNAs were then further filtered through analysis of Fold Change. Hierarchical Clustering was performed to highlight distinguishable circRNAs expression patterns among samples.

### CRC cell lines and cell culture

CRC cell lines including DLD-1, HCT 116, HT 29, LoVo and Caco 2 were purchased from the Cell Bank of Type Culture Collection of the Chinese Academy of Sciences (Shanghai, China), and were cultured and stored according to their instructions. The culture mediums include Dulbecco’s modified Eagle’s medium (DMEM; Winsent, Quebec, Canada), 10% fetal bovine serum, 100 U/ml penicillin, and 100 µg/ml streptomycin. And all the cells were incubated a 5% CO2 humidified incubator at 37 °C.

### RNA isolation and Real-time quantitative PCR

TRIzol reagent was applied to isolate RNA from colonrectal tissues or cell lines according to the manufacturer’s protocol. Then 1 μg RNA was used to synthesize cDNA, followed by analysis of gene expression on ABI 7900 qPCR system. Relative expression was normalized to GAPDH or U6. The detailed primer sequence information was listed in Supplementary Table 3.

### Sanger sequencing

Sanger sequencing was applied to determine the full length of the amplification products. The divergent primers (genechem, Shanghai, China) were designed to confirm the backsplice junction of Hsa_circ_0124554. The distinct product was amplified by outward-facing primers of Hsa_circ_0124554 and was confirmed by Sanger sequencing (Tsingke, Nanjing, China).

### Actinomycin D and RNase R treatment

Transcription was prevented by the addition of 2 mg/ml Actinomycin D or DMSO (Sigma-Aldrich, USA) as the negative control. Total RNA (2 μg) was incubated for 30 min at 37 °Cwith 3 U/μg of RNase R (Sigma-Aldrich, USA). After the treatment, we using qRT-PCR to detected the RNA expression levels of FOXP1 and circFOXP1.

### RNA fluorescence in situ hybridization (FISH)

Fluorescence labeled oligonucleotide probes complementary to hsa_circ_0124554 were designed using the Clone Manager suite of analysis tools. 1 × 10^4^ Cells were seeded on a cover glass-bottom confocal dish and cultured overnight. RNA FISH assay was performed using RNA FISH kit (Suzhou GenePharma Co, Ltd, Suzhou, China) according to manufacturer’s instruction. Nuclei were stained with 4,6-diamidino-2-phenylindole. Images were acquired on ZEISS LSM 880 with Airyscan (Carl Zeiss Microscopy GmbH, Jena, Germany).

### Northern blot

Approximately 10 µg of total RNA was separated in a 1.2% agarose gel containing formaldehyde. The RNA was then transferred to Amersham hybond-N1 membranes (GE Healthcare, Little Chalfont, Buckinghamshire, UK). The membranes were hybridized with digoxin-labeled DNA oligonucleotides specific to hsa_circ_0124554 in Church buffer (0.5 M NaPO4, 7% SDS, 1 mM EDTA, 1% BSA, pH 7.5) at 37 °C and washed in 2× SSC (300 mM NaCl, 30 mM Na-citrate, pH 7.0) with 0.1% SDS at room temperature. The membranes were finally exposed on phosphorimager screens and analyzed using Quantity One or Image Lab software (Bio-Rad, Hercules, CA, USA).

### Plasmid design and lentiviral vectors

The shRNA technology was used to suppress the endogenous expression of hsa_circ_0124554 in cell lines. In shRNA sequence or full length of hsa_circ_0124554 was designed or synthesized and cloned into lentivirus vectors following the manufacturer’s instruction by Genechem (Shanghai, China). Two independent target region was designed for each Forty-eight hours later, puromycin was added to medium for the selection of stable clones. The transfection efficacy was determined by qRT-PCR. The detailed shRNA sequence was listed in Supplementary Table 4.

### Western blot

Proteins were collected from cells and tissues according the manufactory’s protocol (KeyGEN BioTech). In brief, after extraction with RIPA buffer with protease inhibitor and phosphatase inhibitor cocktails (Pierce Biotechnology, Rockford, IL, USA) and quantified with a BCA kit (Thermo Fisher Scientific, Waltham, MA, USA), equal loading proteins of cell lysates or tissue lysates were added to each well of SDS PAGE. Followed by electrophoresising, transfer-membraning, and blocking with 5% non-fat milk in PBST for 1 h, then diluted primary antibodies were incubated at 4 °C overnight.

Protein expression levels were detected by ECL Plus (Millipore, Billerica, MA, USA) with a Bio-Imaging System. The antibody information is listed in Supplementary Table 5.

### Cell proliferation assay

Cell proliferation was measured using CCK-8 assays as previously reported. Briefly, 1 × 10^3^ cells were seeded in 96-well plates and were cultured for 24 h, 48 h, 72 h and 96 h. The absorbance values at 450 nm were then measured using an enzyme immunoassay analyzer (Thermo Fisher Scientific, Inc., Waltham, MA, USA).

### Flow cytometry

Cells were harvested via trypsinization, washed in ice-cold PBS, fixed in ice-cold 75% ethanol in PBS, centrifuged at 4 °C and suspended in PBS. RNase A (Epicenter Technologies, Madison, WI, USA) was then added at a final concentration of 4 mg/ml, followed by incubation at 37 °C for 30 min. Then, 20 mg/ml propidium iodide (Beyotime, Shanghai, China) was added, and the sample was incubated for 20 min at room temperature. The cells were finally analyzed via flow cytometry (BD Biosciences, San Jose, CA, USA).

### Transwell and wound healing assay

Transwell assays and invasion assays were conducted using Millicell cell culture inserts (24-well insert, 8-lm pore size) according to the manufacturer’s instructions. Matrigel (BD Biosciences, Franklin Lakes, NJ, USA) was firstly coated to inserts, and then 8 × 10^4^ cells (per well) in serum-free medium were on. Medium containing 10% FBS was added to the lower chamber. After 24 h (48 h based on different cell lines) of incubation, cells on the bottom of the inserts were fixed and stained with 0.5% crystal violet solution.

Seeding cells into 6-well culture plate at a density of 5 × 10^5^ cells per well. After 24 h incubation, scratch the monolayer with a new 200 ul pipette tip across the center of the well. After scratching, washing the well twice and replenishing the well with fresh medium containing no FBS. Growing cells for another 24 h, photographs were taken to estimate closure of the gap. The gap distance was quantitatively evaluated using software-ImageJ.

### RNA pull-down and mass spectrometry

The biotin-labeled circRNA and the antisense were transcribed with a Biotin RNA Labeling Mix (Roche, CA, USA) and the T7 RNA polymerase (Roche, CA, USA), treated with RNase-free DNase I (Roche, CA, USA) and purified with a RNeasy Mini Kit (Qiagen, Hilden, Germany). Cells were incubated with biotinylated RNAs and 60 μL of streptavidin agarose beads (Invitrogen Life Technologies, CA, USA). The associated proteins were resolved by SDS-PAGE, and specific bands were excised. Proteins were eluted, digested and subjected to the Orbitrap Velos Pro LC/MS system (Thermo Scientific, CA, USA). Data were analyzed by Proteome Discoverer and the resulting peak lists were used for searching the NCBI protein database with the Mascot search engine.

### RNA immunoprecipitation (RIP) assay

RIP assay was performed according to protocols reported previously. The Magna RIP RNA-Binding Protein Immunoprecipitation Kit (Millipore, CA, USA) was employed and conducted according to the manufacturer’s instructions. The IgG antibody were incubated in the magnetic beads’ suspension with rotation for 30 min was negative control. After washing with cold RIP wash buffer, the immunoprecipitated RNA was purified and detected by qRT-PCR.

### In vivo metastasis assays

5- to 6-week-old male BALB/c nude mice were purchased from the animal center of Nanjing University. All animal experiments were performed under the experimental animal use guidelines of the National Institutes of Health. 1 × 10^6^ Cells with different treatment were suspended in 20 μL phosphate-buffered saline (PBS) were injected into the tail vein. The metastasis node was monitored by IVIS ® Spectrum in vivo imaging system every week. The liver metastasis model was established by spleen injection with these transfection groups of CRC cell lines. After 45 days, we sacrificed the mice and evaluated the liver metastasis ability.

### Statistical analysis

Data are presented as mean ± SEM. The *χ*2 tests and the Student’s *t*-test analysis of variances were used to evaluate statistical differences in demographic and clinical characteristics. Pearson correlation analysis was used to analyze the relationship of associated factors. Statistical analysis was performed using STATA10.0. *P* < 0.05 was considered significant.

## Supplementary information

Supplement for review

## Data Availability

The microarray data are available in the ArrayExpress database (www.ebi.ac.uk/arrayexpress) under accession number E-MTAB-8831.
