# Supplementary material for: CircRNA circ_0124554 blocked the ubiquitination of AKT promoting the skip lymphovascular invasion on hepatic metastasis in colorectal cancer
Source: Cell Death Dis. 2021 Mar 15;12(3):270. doi: 10.1038/s41419-021-03565-3 (PMC7960696; doi:10.1038/s41419-021-03565-3)
Supplement: Supplementary file 1 — Supplement for review [file 41419_2021_3565_MOESM1_ESM.pdf]

Supplementary Information

Supplementary Table 1. The clinicopathological characteristics of patients with CRC.

Supplementary Table 2. Correlation of hsa_circ_0124554 expression and clinicopathological characteristics of patients with CRC.

Supplementary Table 3. Primers for Quantitative RT- PCR

Supplementary Table 4. Summary of shRNA Oligos

Supplementary Table 5. Summary of antibodies

Supplementary Figure 1. Hsa_circ_0124554 plays roles in CRC cell lines, but not its linear form. a: qRT-PCR analysis of circFOXP1 and FOXP1 mRNA after treatment with Actinomycin D at the indicated time points in HT29, Caco2 and HCT116 cells. b: Sanger sequencing was applied to determine back-splicing site of circFOXP1. c: The knockdown efficiency of shRNAs for circFOXP1 in HT29 and Caco2 cells. d: The overexpression efficiency of Lv-circRNA for circFOXP1 in HCT116 cell.

Supplementary Figure 2. The linear form control of the RNA was utilized in transwell assay and wound healing assay.

Supplementary Figure 3. Hsa_circ_0124554 could not affect the proliferation, apoptosis or cycle of CRC cells. a: The proliferation of CRC cells was measured by CCK8. b: The cell cycle of CRC cells was detected by flow cytometry. c: The apoptosis of CRC cells was detected by flow cytometry. Data are presented as means ± SD (n.s. indicated no significant).

Supplementary Figure 4. Protein expression of AKT in cells with hsa_circ_0124554 overexpression or suppression. a-b: Representative blot of western blot. c: Statistical analysis of band. Data are presented as means ± SD. *P < 0.05, **P < 0.01, **P < 0.001.

Supplementary Figure 5. The detailed binding site of hsa_circ_0124554 with AKT. a: The detailed bind region in hsa_circ_0124554 and AKT. b: The interaction matrix of binding ability.

Supplementary Figure 6. The phosphorylation of AKT were detected with IGF1

stimulation. a: Representative blot of western blot. b: Representative blot of western blot. Data are presented as means ± SD. *P < 0.05, **P < 0.01, **P < 0.001.

Supplementary Figure 7. Ubiquitination of AKT in CRC cells treated with different groups in the presence of IGF1. a: Representative blot of western blot. b: Statistical analysis of band. Data are presented as means ± SD. **P < 0.01.

Supplementary Figure 8. Correlation between AKT/pAKT and hsa_circ_0124554. a: Relative expression of hsa_circ_0124554 and AKT in cell lines. b: Relative protein expression of AKT and pAKT in cell lines. c: Pearson correlation analysis between AKT and hsa_circ_0124554 and pAKT and hsa_circ_0124554.

Supplementary Table S1. The clinicopathological characteristics of patients with CRC.

		**LNLM1**	**LNLM0**	***p* value[a]**
	**n**	**n=40**	**n=40**	
**Age(years)**				
<60	32	18	14	0.361
≥60	48	22	26	
**Gender**				
Male	50	24	26	0.644
Female	30	16	14	
**Tumor diameter (cm)**				
<5cm	21	12	9	0.446
≥5cm	59	28	31	
**Pathologic type**				
ADC	75	37	38	0.644
MADC	5	3	2	
**Vascular invasion**				
No	21	15	6	**0.022**
Yes	59	25	34	
**T stage**				
T1	10	4	6	0.575
T2	26	15	11	
T3	44	21	23	
**Differentiation degree**				
Highly	10	4	6	0.546
Moderately	58	23	25	
Poorly	12	13	9	
**Primary tumor site**				
Colon	39	21	18	0.502
Rectum	41	19	22	

LNLM1: Lymph node negative with liver metastasis; LNLM0: Lymph node negative without liver metastasis; a: Chi-square test, ADC: Adenocarcinoma; MADC: Mucinous adenocarcinoma

Supplementary Table S2. Correlation of hsa_circ_0124554 expression and clinicopathological characteristics of patients with CRC.

		hsa_circ_0124554 expression[a]		
		**Low**	**High**	***p* value[b]**
	**n**	**n=40**	**n=40**	
**Tumor diameter (cm)**				
<5cm	21	11	10	0.799
≥5cm	59	29	30	
**Pathologic type**				
ADC	75	38	37	0.644
MADC	5	2	3	
**Vascular invasion**				
No	21	16	5	**0.005**
Yes	59	24	35	
**T stage**				
T1	10	5	5	0.885
T2	26	12	14	
T3	44	23	21	
**Differentiation degree**				
Highly	10	5	5	0.818
Moderately	58	28	30	
Poorly	12	7	5	
**Liver metastasis**				
Negative	40	28	12	**0.0003**
Positive	40	12	28	
**Primary tumor site**				
Colon	39	19	20	0.823
Rectum	41	21	20	

a: Medium as cutoff, b: Chi-square test, ADC: Adenocarcinoma; MADC: Mucinous adenocarcinoma

Supplementary Table S3. Primers for Quantitative RT- PCR

Gene name	All Patients	Sequence
Circ_0124554	Forward Primer	TGCAGAAGAAACCACAGGCA
	Reverse Primer	CTTGGAAGGTGCAGAGGAGG
Circ_0097743	Forward Primer	GGTTAAGGGGCAGATCCCAA
	Reverse Primer	GCGGCCAAGAAGCGATTTAG
Circ_0079875	Forward Primer	TGGGTTCTGATGCCAGTCTT
	Reverse Primer	GGCCACGAAGACATGCTAGT
Circ_0009582	Forward Primer	GAGTGGTGTCATCCCGAGAG
	Reverse Primer	GGGAACTGGTTCATCATGCG
EGFR	Forward Primer	GACGACAGGCCACCTCG
	Reverse Primer	CCCATTGGGACAGCTTGGAT
GAPDH	Forward Primer	GGAGCGAGATCCCTCCAAAAT
	Reverse Primer	GGCTGTTGTCATACTTCTCATGG

Supplementary Table S4. Summary of shRNA Oligos

Name	Oligo Sequence
hsa_circ_0124554 shRNA-1	CACCGCCAAGGCCTTCTGACAATTCCGAAGAATT GTCAGAAGGCCTTGGC
	AAAAGCCAAGGCCTTCTGACAATTCTTCGGAATT GTCAGAAGGCCTTGGC
hsa_circ_0124554 shRNA-2	CACCACCTCTTGCTCAAGGCATGATCGAAATCAT GCCTTGAGCAAGAGG
	AAAACCTCTTGCTCAAGGCATGATTTCGATCATG CCTTGAGCAAGAGGT
hsa_circ_0124554 shRNA-3	CACCGCAGTTTGGATCTGACCACGACGAATCGTG GTCAGATCCAAACTGC

Supplementary Table S5. Summary of antibodies

Name	Catalog#	Company	Application
Anti-AKT antibody	ab182729	Abcam	WB, IHC, IP
Anti-AKT1 (phospho S473) antibody	ab81283	Abcam	WB
Phospho-Akt (Thr308) (D25E6) XP	Cst13038	CST	WB
Anti-GAPDH antibody [6C5]	ab8245	Abcam	WB

WB: Western blot

IHC: immunohistochemistry

IP: immunoprecipitation

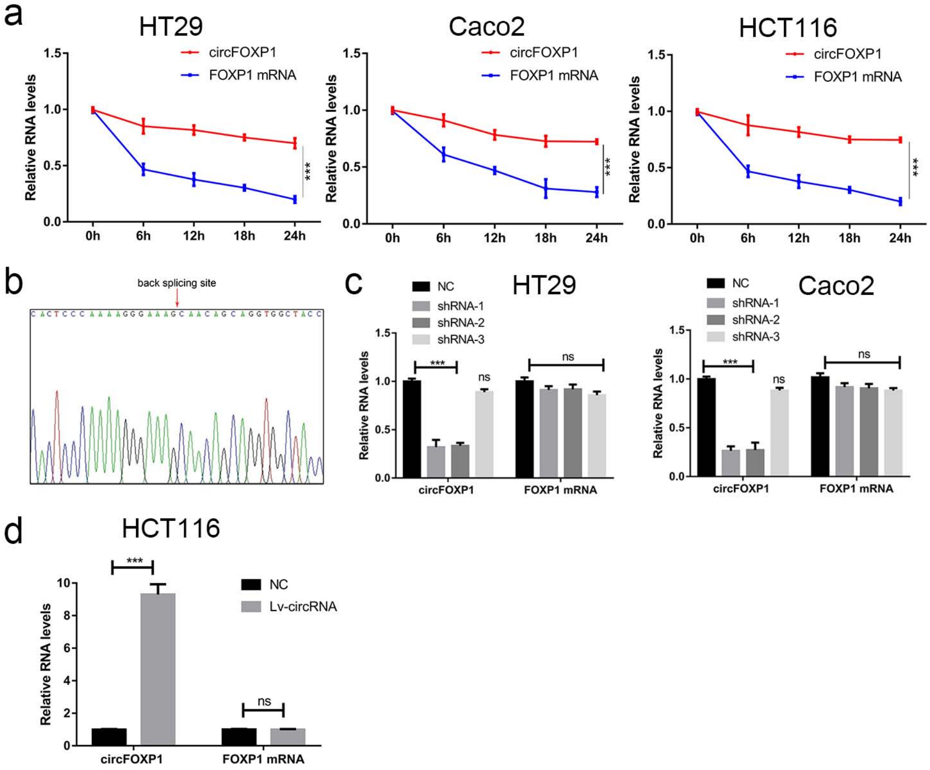
a
HT29
Caco2
HCT116
circFOXP1
FOXP1 mRNA
Relative RNA levels
0h
6h
12h
18h
24h
***
b
back splicing site
CACTCCCAAAAGGGAAAGCAACAGCAGGTGGCTACC
c
HT29
Caco2
NC
shRNA-1
shRNA-2
shRNA-3
ns
circFOXP1
FOXP1 mRNA
d
HCT116
NC
Lv-circRNA

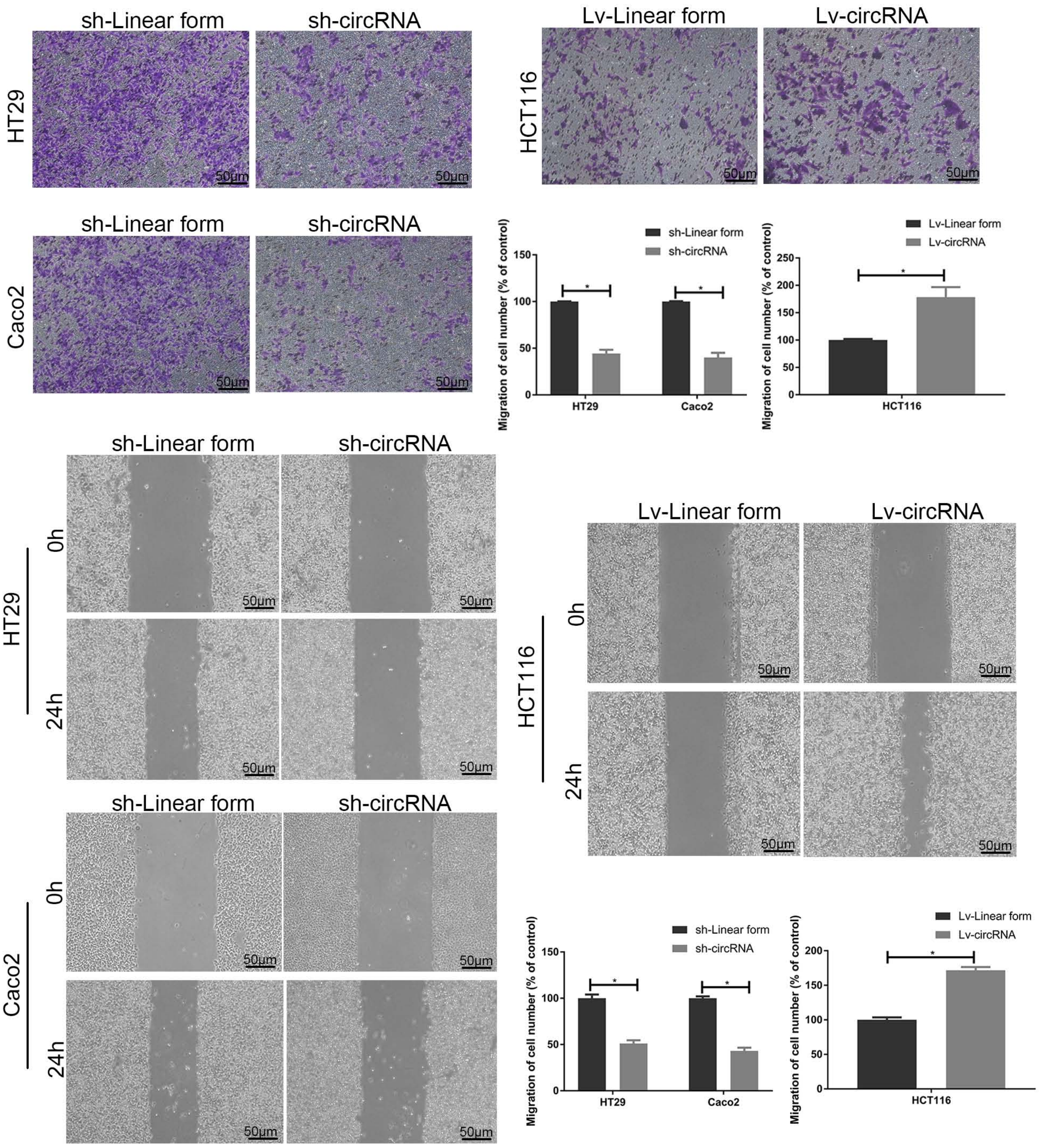
sh-Linear form
sh-circRNA
HT29
Caco2
50μm
Lv-Linear form
Lv-circRNA
HCT116
Migration of cell number (% of control)
sh-Linear form
sh-circRNA
150
100
50
0
HT29
Caco2
*
Lv-Linear form
Lv-circRNA
250
200
150
100
50
0
HCT116
0h
24h

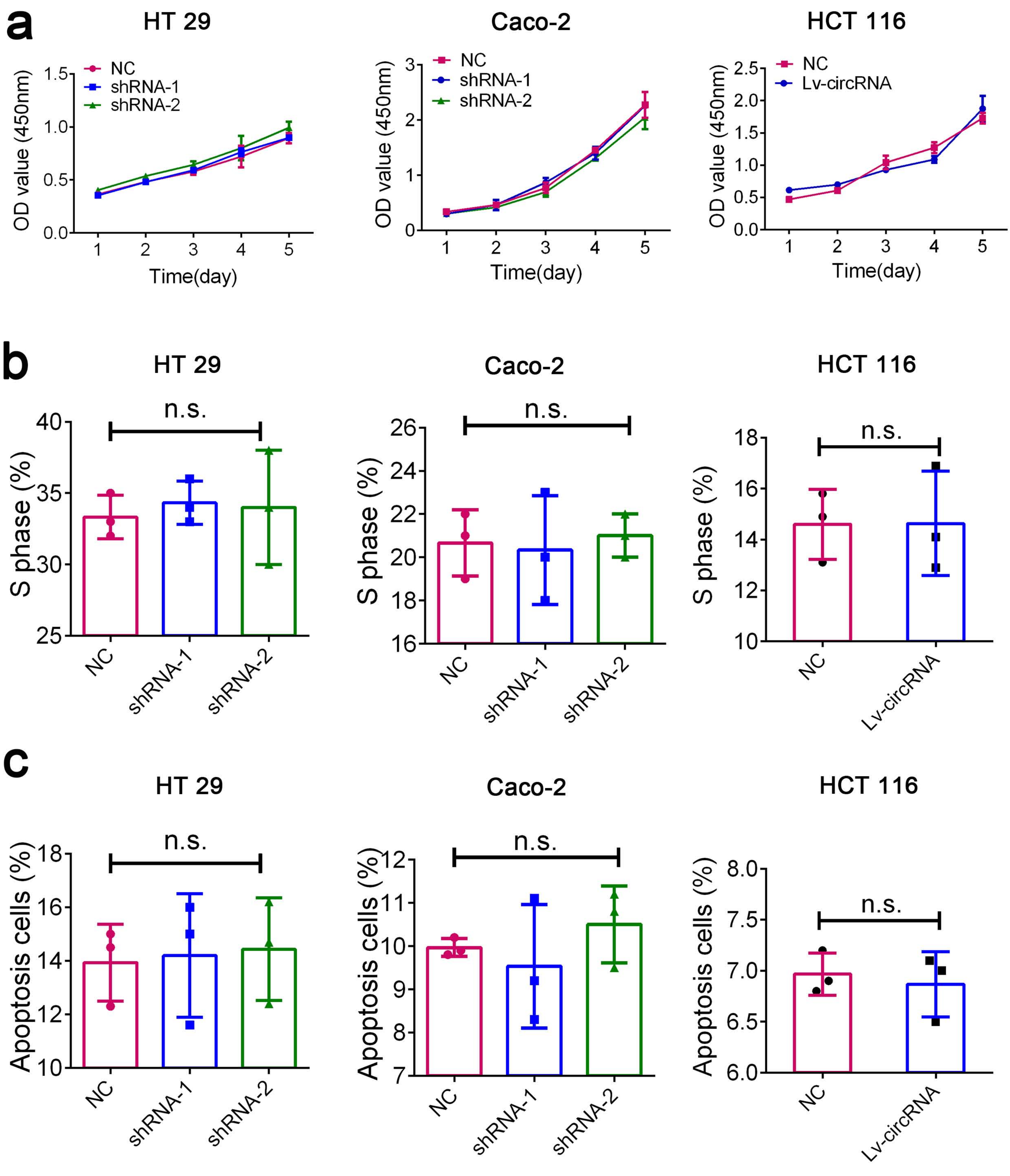

a
HT 29
OD value (450nm)
Time(day)
NC
shRNA-1
shRNA-2
Caco-2
HCT 116
Lv-circRNA
b
S phase (%)
n.s.
c
Apoptosis cells (%)

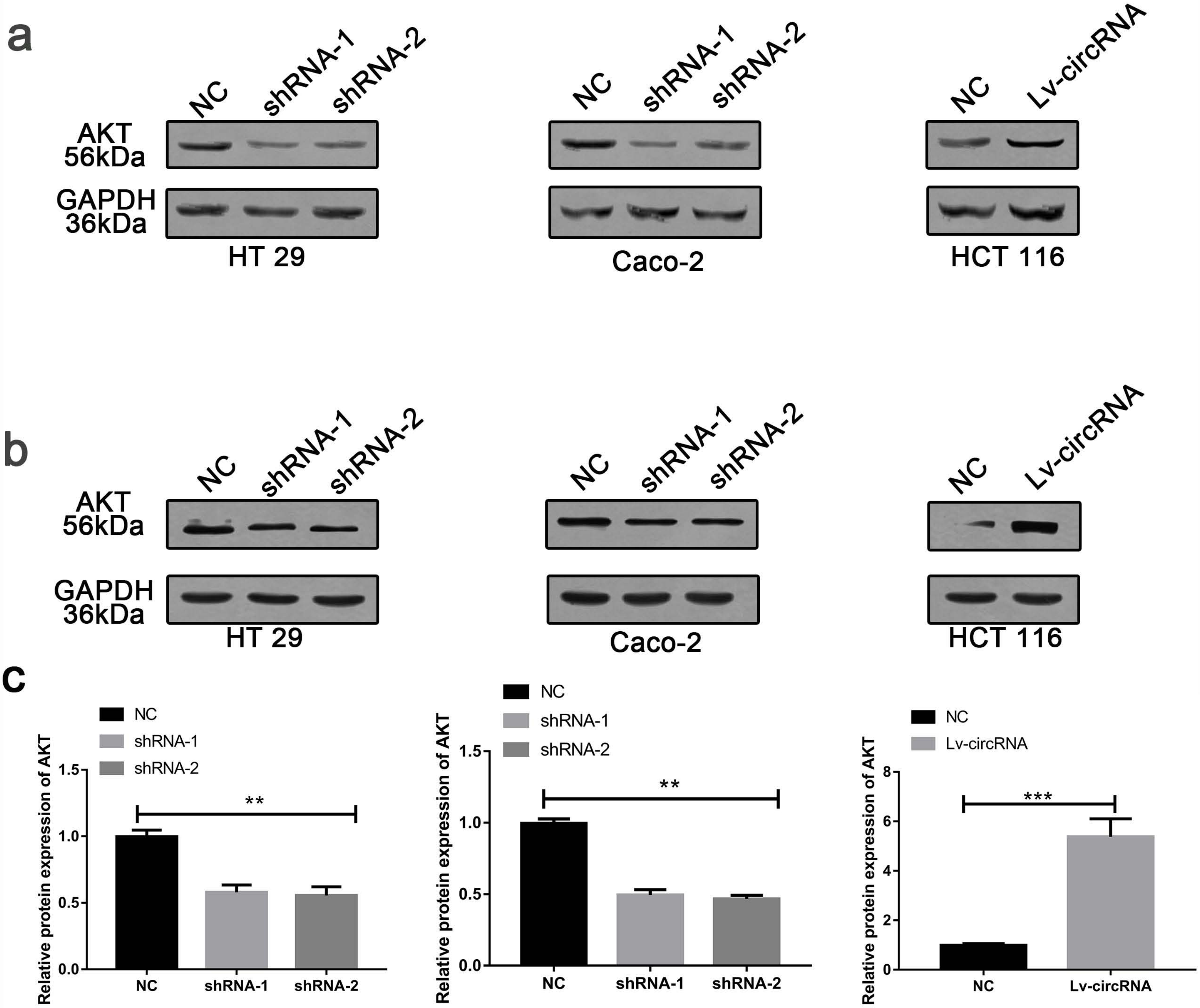
a
NC
shRNA-1
shRNA-2
AKT
56kDa
GAPDH
36kDa
HT 29
Caco-2
Lv-circRNA
HCT 116
b
c
Relative protein expression of AKT
1.5
1.0
0.5
0.0
**
***
8
6
4
2
0

a

#	Protein region	RNA region	Interaction Propensity	Discriminative Power	Normalized Score
1	01- 52	76-127	7.44	24	2.60
2	10 -52	76-127	6.93	22	2.46
3	180-231	76-127	6.87	22	2.44
4	355-406	76-127	6.59	22	2.36
5	276-327	76-127	6.57	22	2.35
6	176-227	76-127	6.50	22	2.33
7	105-156	76-127	6.14	22	2.23
8	351-402	51-102	5.48	22	2.05
9	01- 52	84-135	5.43	22	2.03
10	101-152	51-102	5.24	22	1.98
11	351-402	76-127	5.17	22	1.96
13	80-131	284-335	5.16	22	1.96
12	280-331	76-127	5.17	22	1.96
15	180-231	51-102	5.03	22	1.92
14	180-231	84-135	5.04	22	1.92
16	130-181	76-127	4.95	20	1.90
17	101-152	84-135	4.89	20	1.88
18	276-327	51-102	4.85	20	1.87
19	276-327	84-135	4.84	20	1.87
20	10 -52	84-135	4.82	20	1.86

b

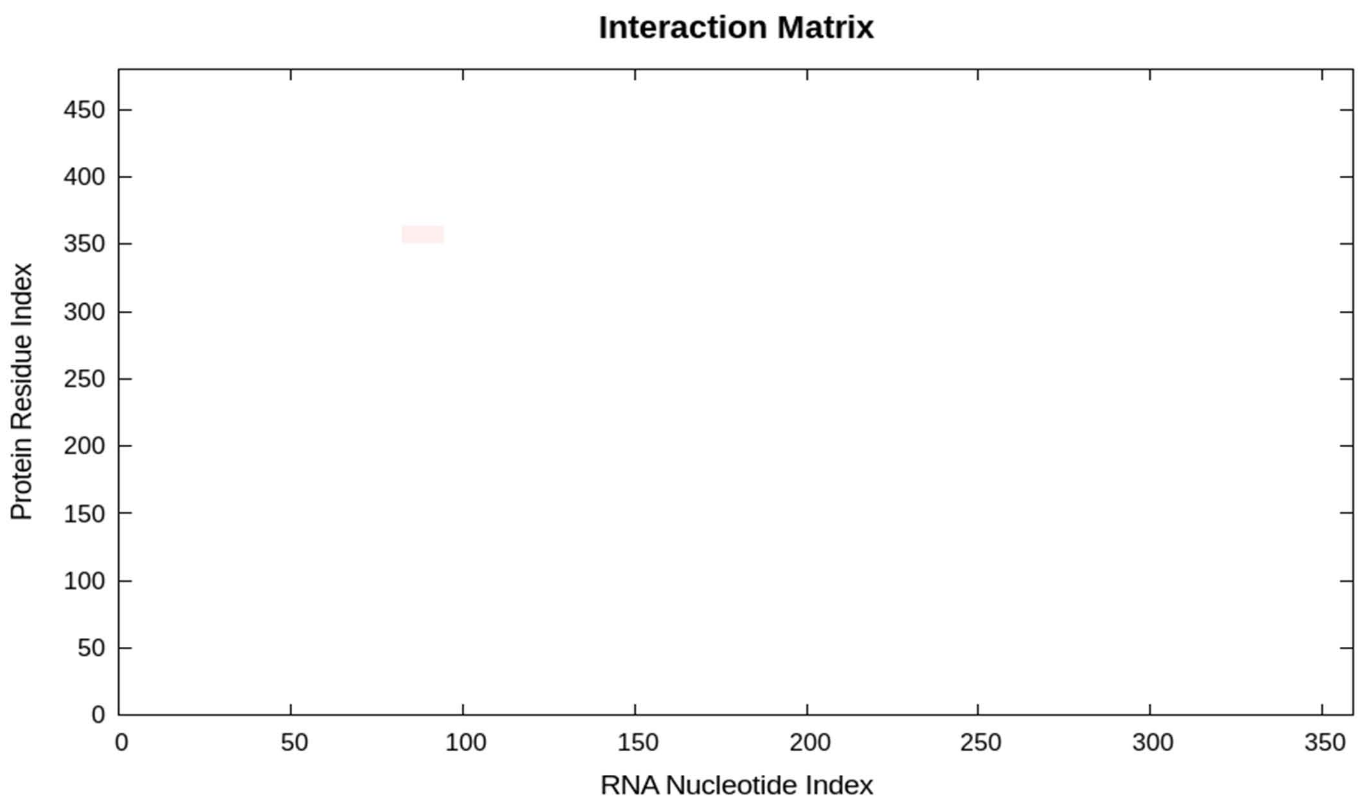

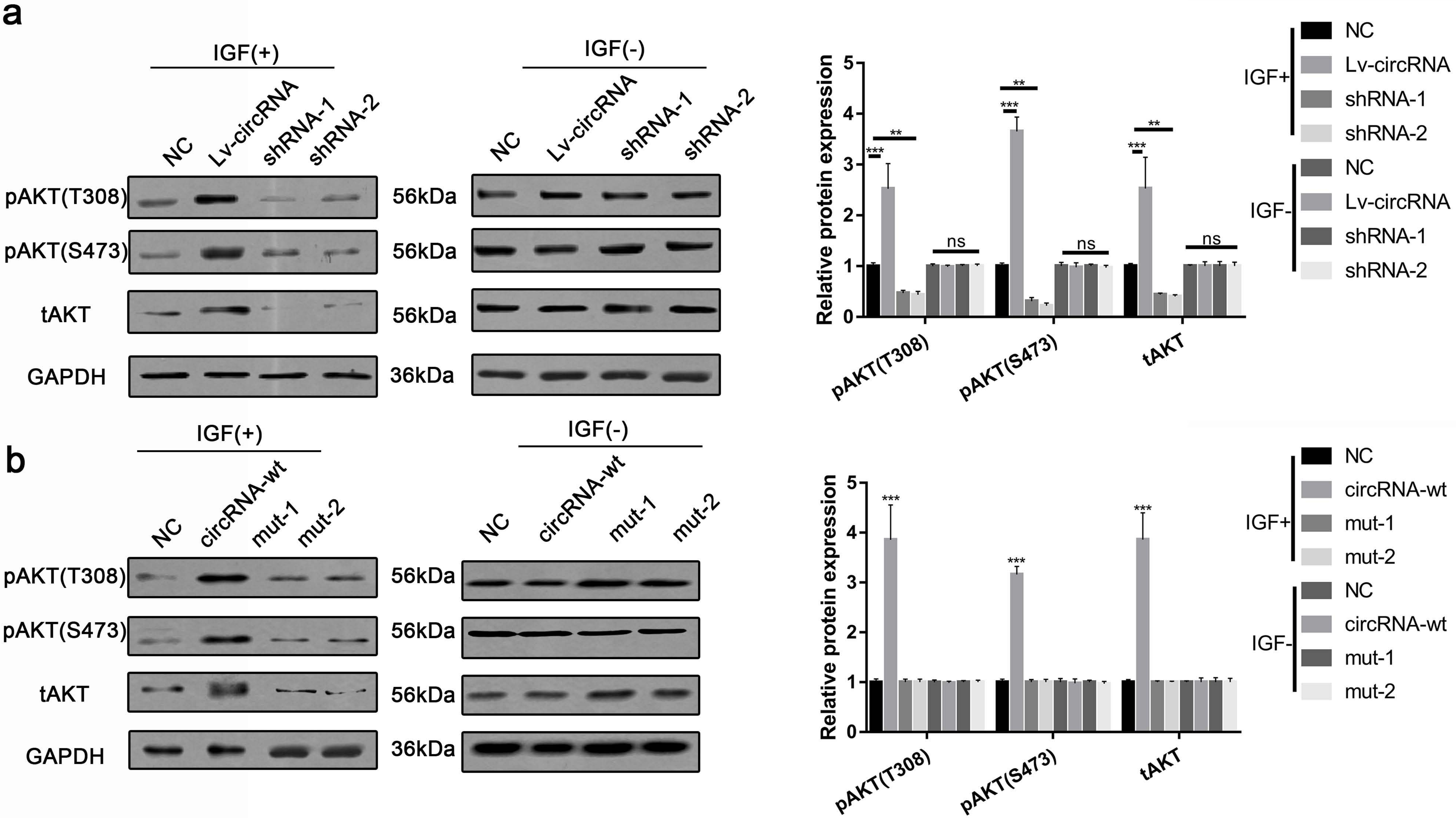
a
IGF(+)
NC
Lv-circRNA
shRNA-1
shRNA-2
IGF(-)
NC
Lv-circRNA
shRNA-1
shRNA-2
pAKT(T308)
pAKT(S473)
tAKT
GAPDH
56kDa
56kDa
56kDa
36kDa
Relative protein expression
0
1
2
3
4
5
***
**
ns
pAKT(T308)
pAKT(S473)
tAKT
IGF+
NC
Lv-circRNA
shRNA-1
shRNA-2
IGF-
NC
Lv-circRNA
shRNA-1
shRNA-2
b
IGF(+)
NC
circRNA-wt
mut-1
mut-2
IGF(-)
NC
circRNA-wt
mut-1
mut-2
pAKT(T308)
pAKT(S473)
tAKT
GAPDH
56kDa
56kDa
56kDa
36kDa
Relative protein expression
0
1
2
3
4
5
***
pAKT(T308)
pAKT(S473)
tAKT
IGF+
NC
circRNA-wt
mut-1
mut-2
IGF-
NC
circRNA-wt
mut-1
mut-2

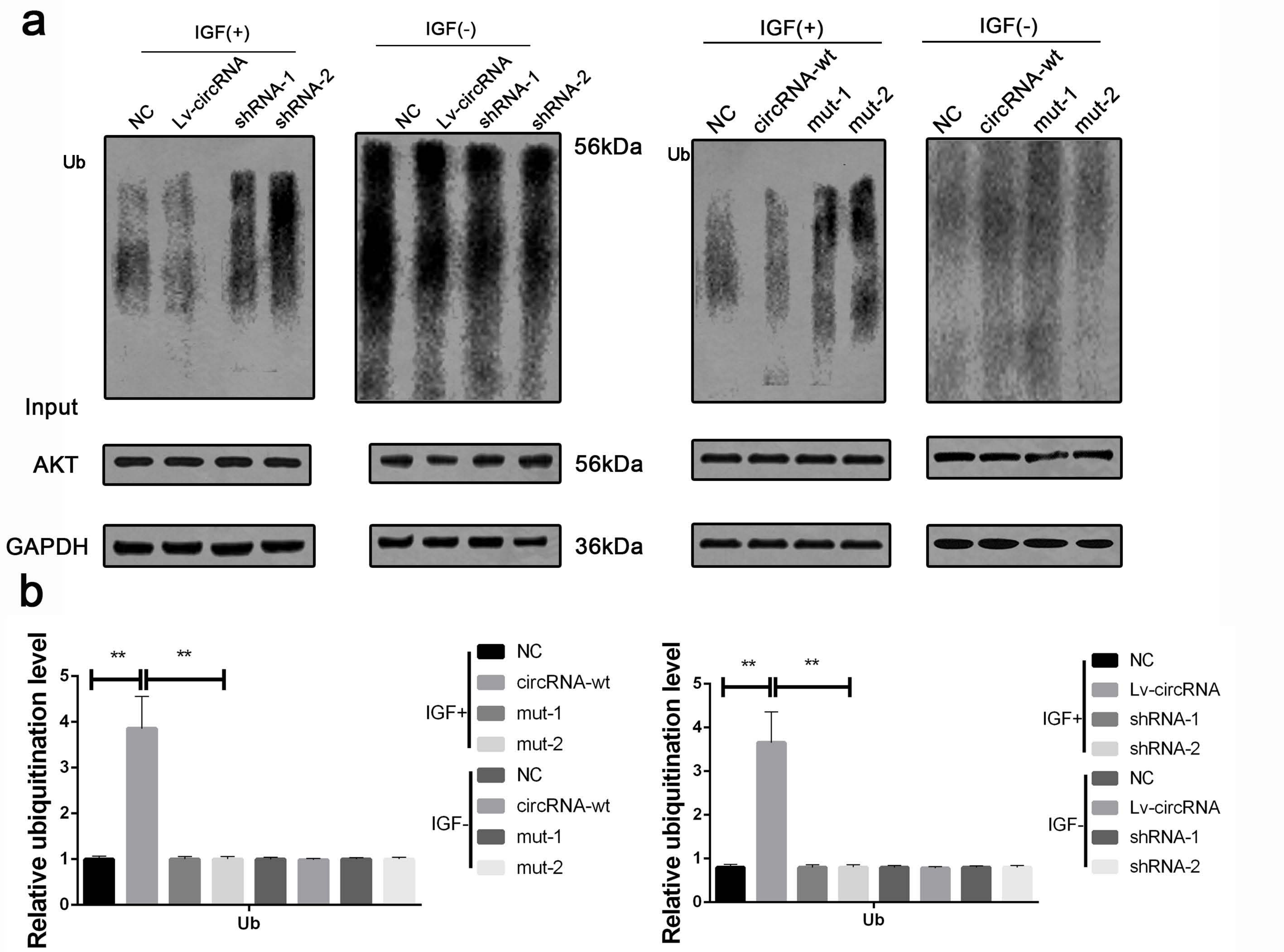

a
IGF(+)
IGF(-)
NC
Lv-circRNA
shRNA-1
shRNA-2
circRNA-wt
mut-1
mut-2
Ub
56kDa
Input
AKT
56kDa
GAPDH
36kDa
b
Relative ubiquitination level
**
**
Ub
NC
circRNA-wt
mut-1
mut-2
IGF+
IGF-
NC
Lv-circRNA
shRNA-1
shRNA-2

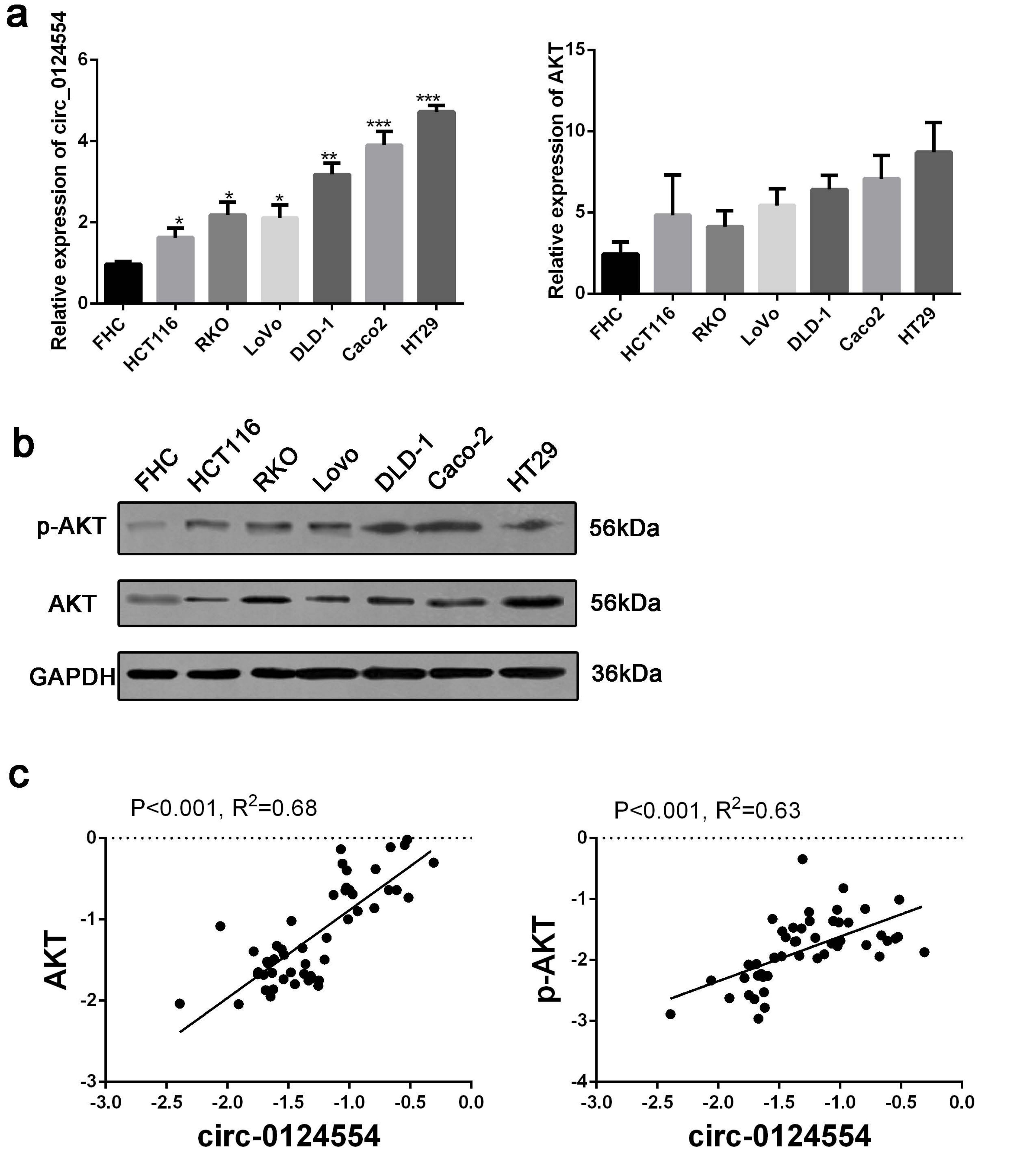
a
Relative expression of circ_0124554
6
4
2
0
*
*
*
**
***
***
FHC
HCT116
RKO
LoVo
DLD-1
Caco2
HT29
Relative expression of AKT
15
10
5
0
FHC
HCT116
RKO
LoVo
DLD-1
Caco2
HT29
b
FHC
HCT116
RKO
Lovo
DLD-1
Caco-2
HT29
p-AKT
56kDa
AKT
56kDa
GAPDH
36kDa
c
P<0.001, R2=0.68
AKT
0
-1
-2
-3
-3.0
-2.5
-2.0
-1.5
-1.0
-0.5
0.0
circ-0124554
P<0.001, R2=0.63
p-AKT
0
-1
-2
-3
-4
-3.0
-2.5
-2.0
-1.5
-1.0
-0.5
0.0
circ-0124554
